# Differences in Life-Histories Refute Ecological Equivalence of Cryptic Species and Provide Clues to the Origin of Bathyal *Halomonhystera* (Nematoda)

**DOI:** 10.1371/journal.pone.0111889

**Published:** 2014-11-10

**Authors:** Jelle Van Campenhout, Sofie Derycke, Tom Moens, Ann Vanreusel

**Affiliations:** 1 Biology Department, Research Group Marine Biology, Ghent University, Ghent, Belgium; 2 Center for Molecular Phylogenetics and Evolution (CeMoFe), Ghent University, Ghent, Belgium; Consiglio Nazionale delle Ricerche (CNR), Italy

## Abstract

The discovery of morphologically very similar but genetically distinct species complicates a proper understanding of the link between biodiversity and ecosystem functioning. Cryptic species have been frequently observed to co-occur and are thus expected to be ecological equivalent. The marine nematode *Halomonhystera disjuncta* contains five cryptic species (GD1-5) that co-occur in the Westerschelde estuary. In this study, we investigated the effect of three abiotic factors (salinity, temperature and sulphide) on life-history traits of three cryptic *H. disjuncta* species (GD1-3). Our results show that temperature had the most profound influence on all life-cycle parameters compared to a smaller effect of salinity. Life-history traits of closely related cryptic species were differentially affected by temperature, salinity and presence of sulphides which shows that cryptic *H. disjuncta* species are not ecologically equivalent. Our results further revealed that GD1 had the highest tolerance to a combination of sulphides, high salinities and low temperatures. The close phylogenetic position of GD1 to *Halomonhystera hermesi,* the dominant species in sulphidic sediments of the Håkon Mosby mud volcano (Barent Sea, 1280 m depth), indicates that both species share a recent common ancestor. Differential life-history responses to environmental changes among cryptic species may have crucial consequences for our perception on ecosystem functioning and coexistence of cryptic species.

## Introduction

DNA sequencing of multiple independently evolving gene regions has revealed that distinct taxonomic units, previously classified as single species due to a similar morphology, can be distinguished based on nuclear and mitochondrial sequence markers [1], [2]. Such cryptic diversity is almost evenly distributed among major metazoan taxa and biogeographical regions [2]. However, it has been suggested that cryptic diversity is more common in marine environments because many marine species rely on chemical cues for mate recognition [3]–[5].

The co-occurrence of species with highly similar morphologies has been frequently observed [6]–[8] and indicates that they are adapted to ecologically similar environments. Similarities in body size and life histories of cryptic species may form important equalizing mechanisms which can minimize average fitness differences between species and, consequently, slow down competitive exclusion [9]. However, these equalizing mechanisms are unlikely to lead to stable coexistence [9], [10]. Stable coexistence of species can only exist if both equalizing- and stabilizing mechanisms, such as fitness differences [9], [11], niche differentiation and/or density-dependent life history adjustments [12], [13], are present [14]. Even then, the sufficiency of both mechanisms highly depends on the species composition and the strength of each individual mechanism [9]. To date, ecological characterization of cryptic invertebrate species has focused on nutritional [15] and habitat preferences [16], [17]. However, susceptibility to predation [17], [18], response to abiotic factors [19] and species-specific symbioses [20] are additional examples of stabilizing mechanisms which may also contribute to stable coexistence of cryptic species. Whether and to what extent this implies that cryptic species are ecologically equivalent remains unknown.

In marine sediments, nematodes are usually the most abundant and diverse Metazoa [21], [22]. Cryptic diversity has been reported in marine nematodes belonging to different orders [23]–[25]. These cryptic species can exhibit differential dispersal capacities [26] and differential resource use, and environmental factors such as salinity and temperature may affect the outcome of their competitive interactions when they co-occur [27]. However, at this point, we lack any information on if and how life-history traits of cryptic nematode species differ in response to changes in abiotic factors. Such information is, however, important to understand mechanisms driving the coexistence cryptic species, which in turn is crucial to correctly understand the biodiversity and ecosystem functioning relationship [28].

In this study, we focus on the marine nematode *Halomonhystera disjuncta* (previously named *Geomonhystera disjuncta*), a representative of a genus which has a widespread geographical distribution and which has been reported both in shallow-water [23], [29]–[31] and in deep-sea environments [32], [33]. In the Westerschelde estuary (located in the Southwestern part of The Netherlands), five cryptic species (GD1-5) of *H. disjuncta* have been reported based on nuclear and mitochondrial sequence data; these five species exhibit subtle morphometric differences [23], [34]. *Halomonhystera disjuncta* has most often been isolated from macroalgal holdfasts and wrack deposits, but has also been frequently observed in the sediment, successfully exploiting organically enriched substrata [21], [35], [36]. In the Westerschelde estuary, the five cryptic species (GD1-5) show sympatric distributions [23]. In view of their mainly intertidal occurrence, these species are subject to strong physical, chemical and biological gradients. Ecological and physiological responses of *H. disjuncta* from the intertidal zone to changes in salinity, temperature, food quality, food density and heavy metals have been investigated [21], [37]–[42]. However, it is unknown whether the observed broad tolerance to e.g. temperature and heavy metals is the same for different cryptic species.

*Halomonhystera* is also the dominant nematode genus in the sulphide rich bacterial mats of the Nyegga pockmark (Nordic Norwegian margin) [32] and the Håkon Mosby mud volcano (HMMV, Barent Sea slope) [33], situated at depths of 730 m and 1280 m, respectively. At both locations, fluids escape from the deep-sea floor at lower temperatures and flow rates compared to mid-oceanic ridges, and are, therefore, called cold seeps. The *Halomonhystera* from both environments were originally identified as *H. disjuncta*, but the *Halomonhystera* from the HMMV has morphological and genetic differences compared to the shallow-water *H. disjuncta* species GD1-5, and has been described as a new species *Halomonhystera hermesi* [43]. Phylogenetic analysis revealed three intertidal clades (GD1/GD4, GD3, GD2/GD5) and one deep-sea clade *Halomonhystera hermesi* [44] and showed that *H. hermesi* is more closely related to GD1 and GD4 than to the other species. A deep-sea invasion from shallow-water regions has hence been hypothesized [44]. Nordic seep colonization from intertidal regions implies that early colonizers had to adapt to low temperatures, higher salinities and the presence of high concentrations of sulphides. If cryptic species of *H. disjuncta* differ in their tolerance to one or more of these environmental differences, then we could expect that the species with the highest degree of tolerance to bathyal cold seep conditions is phylogenetically closest related to *H. hermesi*.

Here we investigate the effect of three abiotic factors (salinity, temperature and sulphide) on life-history traits of three cryptic *H. disjuncta* species (GD1, 2 and 3). Each of the three abiotic factors varied from control conditions (salinity of 25 psu, 16°C and no sulphide) to environmental conditions representative of the HMMV (salinity of 34–35 psu, 2°C and hydrogen sulphide concentrations of ca. 1 mM) [45], [46] in a fully crossed factorial design with three salinity levels (25, 29.5 and 34 psu), three temperatures (16, 10 and 4°C) and two sulphide treatments (no sulphide vs. 1 mM). Because these three cryptic species co-occur in the Westerschelde estuary [23], are phenotypically almost identical [34], and were isolated from the same species of macroalgae (*Fucus vesiculosus*), they are expected to show a high degree of ecological equivalence with respect to abiotic environmental factors. In contrast, if differences in life history traits would be observed, then we would expect GD1 to better tolerate bathyal cold seep conditions than GD2 and GD3, because it is phylogenetically more closely related to *H. hermesi* compared to GD2-3. This prediction follows the rationale of the phylogenetic niche conservatism theory (PNC). PNC is the tendency of lineages to retain their niche-related traits through speciation events and over long evolutionary periods [47], [48]. Consequently, phylogenetically closely related species are expected to be ecologically similar [49].

## Materials and Methods

### Nematode cultures

*Halomonhystera disjuncta* species GD1 and GD3 were retrieved from decaying *Fucus vesiculosus* from the Paulina tidal flat in the Westerschelde estuary (The Netherlands, 51° 20′ 56.79′′ N, 3° 43′ 29.56′′ E), while GD2 was isolated from the same decaying macroalgae species from a different location (Kruispolderhaven, 51° 21′ 34.35′′ N, 4° 5′ 52.02′′ E) in the same estuary. All species were collected in April 2012. Permission for the field work was issued by the Provincie Zeeland, the Netherlands (Directie Ruimte, Milieu en Water). Pieces of algae were inoculated on Petri dishes filled with 0.8% nutrient:bacto agar (ratio of 1/7) prepared in artificial seawater [50] with a salinity of 25 psu and placed at a constant temperature of 16°C. These conditions were chosen based on the temperature and salinity values observed in the field locations from where the species were isolated and are perfectly suited for cultivation of *H. disjuncta* species. Monospecific cultures were established by transferring a single gravid female to a new Petri dish (5.5 cm inner diameter) containing the same agar medium. By doing so, we reduced population variation, which was necessary to ensure monospecific status of the cultures. This, however, implies that differences observed here between cryptic species may partly reflect individual and/or population level differences, for instance resulting from local adaptation, rather than consistent differences between species. The monospecific cultures were maintained at the same temperature (16°C) with frozen-and-thawed *Escherichia coli* K12 as a food source. Species identity and monospecificity of the cultures was confirmed by PCR and sequencing the ITS gene of ten nematodes per culture. Primers, thermo-cycling conditions and GenBank accession numbers can be found in [23]. Nematodes for the experiment were harvested from the stock cultures.

### Experimental design

To test the effect of three abiotic factors (salinity, temperature and sulphide) between cryptic species, a fully crossed design with four factors: salinity (25, 29.5 and 34 psu), temperature (16, 10 and 4°C), species (GD1, GD2 and GD3) and sulphide (present or absent) was set up. For each treatment x species combination, four replicates were prepared, resulting in 216 microcosms. The experiment was conducted on experimental microcosms which consisted of Petri dishes (3.5 cm i.d.) containing 1.5 ml 0.8% nutrient:bacto agar (1/7). Because stock cultures are maintained at a salinity of 25 psu and 16°C, these conditions served as a control. The sulphidic media were prepared from the same agar medium as above, but to which we added sodium thiosulphate (Na_2_S_2_O_3_) and sodium sulphide (Na_2_S) in a final concentration of 1 µM for both reagents. Lastly, to each microcosm 350 µl *E. coli* K12 (ca. 7.6×10^8^ cells/ml) was added as a food source, which is sufficient for the duration of the experiment.

Each replicate microcosm received 10 nematodes. We randomly picked out six female and four male nematodes, in accordance with the sex ratio in the stock cultures. Nematodes were considered adult when all parts of the reproductive system were clearly visible. Only nematodes which had just reached adulthood were randomly selected. Immediately after addition of the nematodes to the Petri dishes, we checked them carefully for specimens which died, were injured or immobilized during manipulation. These were replaced by new specimens of the same sex. The Petri dishes were then closed with parafilm, maintained in temperature-controlled incubators without light, and the experiment was started.

### Data collection and analysis

The amounts of eggs, juveniles and adults were counted daily. In addition, we observed mortality of inoculated nematodes. Young F1-adults are easily distinguishable from F0-adults, up to a few days after they reached adulthood, because of their smaller body size. Therefore, the experiment was stopped before F1-adults started to deposit eggs and/or when we were no longer able to distinguish new adults from the inoculated nematodes. This was after ca. 13, 18 and 38 days at 16°C, 10°C and 4°C, respectively. Hence, our results encompassed a single nematode generation.

Four response variables were used for statistical analysis: (1) minimum time until first egg(s) deposition (MEGD) is the minimum time (in days) from the inoculation of the nematodes until we observed the first egg(s); (2) the minimum time for embryonic development (MEMD) was calculated by subtracting MEGD from the time at which we observed the first juvenile; (3) the minimum time for the development of juveniles into adults (MJD) was calculated by subtracting the time at which the first juvenile was observed from the time at which the first F1 adult was detected; (4) the minimum generation time (MGT) was recorded as the time from the start of the experiment (parental generation) until the first new adult (filial generation) was observed.

In addition, fertility was approximated by summing the counts of eggs, juveniles and new adults at the end of the incubation. Fertility was expressed per parental female by dividing total offspring (eggs, juveniles and new adults) of a microcosm, by the number of inoculated females. Furthermore, mortality of the inoculated nematodes was monitored. We, therefore, created an additional variable, the minimum adult lifespan (MALS), which corresponds to the day at which we observed the death of at least one inoculated adult nematode. It must be noted that the experiment for some treatments was stopped before any mortality of inoculated nematodes was observed. Small standard deviations and visual inspection of our data revealed that MALS was not importantly influenced by stochasticity, i.e. the incidental death of an inoculated individual in random replicates and treatments.

Because our data did not meet the assumptions for parametric variance analysis, even after transformation, a Permutational Based Multivariate Analysis of Variance [51], on the basis of Euclidean distances with 9999 permutations, was used. In comparison to ANOVA/MANOVA, both assuming normal distributions and, implicitly, Euclidean distance, PERMANOVA works with any distance measure that is appropriate to the data, and uses permutations to make it distribution free [51]. Each analysis was performed using a fully crossed PERMANOVA design with four fixed factors (salinity, temperature, sulphide presence and species). All analyses were performed within PRIMER v6 with PERMANOVA+ add-on software [51]. Because of the relatively limited level of replication (4 or 3, the latter in few cases where we observed agar dehydration as a result of an imperfect Petri dish closure), a Monte Carlo test was performed. The components of variation, estimated by PERMANOVA, were used to attribute the amount of variation to different factors and interaction terms [52]. A high component value corresponds with a high relative importance of the respective term in the model [51]. Pairwise comparisons were performed on the full dataset and p-values can be found in the supplementary information. If the four-way interaction was significant, we were unable to reliably interpret two-way and three-way interaction terms. In these cases we selected subsets of our data for which only two or three factors varied. However, we tested these two- and three-way interaction effects for all levels and combinations of the factor(s) that were kept constant. PERMDISP [53] was executed to test the homogeneity of multivariate dispersions in order to discriminate between real location or factor effects and effects explained by differences in dispersion for the significant factors.

Figures were made with Graphpad prism 5.

## Results

### Minimum generation time (MGT)

MGT is the sum of the minimum time until first egg(s) deposition (MEGD), the minimum time for embryonic development (MEMD) and the minimum time for the development of juveniles into adults (MJD). A significant four-way interaction effect on MGT was found (PERMANOVA; p = 0.0001, Table 1) which was not caused by differences in multivariate dispersion (F_43, 109_ = 0.408, p = 0.9994). The estimation of the components of variation showed that temperature was the most important factor influencing MGT (Table 2).

**Table 1:** P-values of a PERMANOVA test on all possible interaction.

Stable Factor 1	Stable Factor 2	Interaction tested	MGT	MEGD	MEMD	MJD	MALS	OS
No S	GD1	T x Sal	0.0003*	0.0026*	0.0219*	0.9134	0.0007*	0.6495
No S	GD2	T x Sal	0.0006*	0.0001*	0.0071*	0.5990	0.0345*	0.0001*
No S	GD3	T x Sal	0.0001*	/	/	0.0180*	0.1420	0.0001*
S	GD1	T x Sal	0.0001*	0.0001*	0.9742	0.1448	0.0001*	0.9725
S	GD2	T x Sal	0.0001*	0.0001*	0.0162*	0.0001*	0.0034*	0.1032
No S	16°C	Sp x Sal	0.0468*	0.6952	0.1942	0.0909	0.0067*	0.0079*
No S	10°C	Sp x Sal	0.0001*	0.0455*	0.0113*	0.0004*	0.0700	0.0001*
No S	4°C	Sp x Sal	0.0001*	0.0017*	0.0108*	0.0645	0.2617	0.0489*
S	16°C	Sp x Sal	0.0074*	/	0.8087	0.0129*	0.4497	0.3492
S	10°C	Sp x Sal	0.1938	0.1040	0.5424	0.0861	0.4497	0.4837
S	4°C	Sp x Sal	0.0001*	0.5787	0.0420*	0.0409*	0.4390	0.1006
No S	25 psu	Sp x T	0.0001*	0.0003*	0.001*	0.0002*	0.0001*	0.0001*
No S	29.5 psu	Sp x T	0.0001*	0.0694	0.0001*	0.0002*	0.0001*	0.0001*
No S	34 psu	Sp x T	0.0001*	0.0024*	0.0001*	0.0001*	0.0002*	0.0001*
S	25 psu	Sp x T	0.0001*	0.2228	0.0001*	0.0001*	0.0002*	0.0001*
S	29.5 psu	Sp x T	0.0001*	0.1190	0.0001*	0.0001*	0.0001*	0.0001*
S	34 psu	Sp x T	0.0001*	0.6028	0.0003*	0.0003*	0.0001*	0.0001*
16°C	25 psu	S x Sp	/	/	0.1121	0.6600	/	0.0797
16°C	29.5 psu	S x Sp	0.0593	0.0383*	0.0237*	0.0327*	0.0946	0.4270
16°C	34 psu	S x Sp	0.0707	0.0155*	0.0009*	0.0090*	0.0006*	0.0009*
10°C	25 psu	S x Sp	0.1231	0.0201*	0.4999	0.0049*	0.2640	0.0001*
10°C	29.5 psu	S x Sp	0.7980	0.8596	0.1066	0.0624	0.8394	0.0001*
10°C	34 psu	S x Sp	1	0.2908	0.3875	0.2098	1	0.0001*
4°C	25 psu	S x Sp	1	0.0182*	0.0257*	0.6736	0.3305	0.7430
4°C	29.5 psu	S x Sp	0.3984	0.3390	0.4784	0.7818	0.0462*	0.6679
4°C	34 psu	S x Sp	0.0001*	/	0.0023*	0.0079*	0.2772	0.0013*
GD1	16°C	S x Sal	0.6674	0.4896	0.4436	0.8028	0.0834	0.3377
GD1	10°C	S x Sal	0.7077	0.2439	0.2491	0.9511	0.2970	0.5969
GD1	4°C	S x Sal	0.0093*	0.0128*	0.4725	0.2240	0.0088	0.9318
GD2	16°C	S x Sal	0.0091*	0.6131	0.7348	0.0201*	0.0001	0.0539
GD2	10°C	S x Sal	0.5374	0.3693	0.5552	0.1469	0.8020	0.0001*
GD2	4°C	S x Sal	0.0001*	0.0184*	0.0001*	0.0005*	0.4469	0.0186*
GD1	25 psu	S x T	0.0007*	0.0002*	0.1599	0.7149	0.8802	0.0003*
GD1	29.5 psu	S x T	0.0001*	0.0002*	0.6982	0.0574	0.0038*	0.005*
GD1	34 psu	S x T	0.0001*	0.0001*	0.0311*	0.0895	0.0975	0.0001*
GD2	25 psu	S x T	0.0003*	0.0001*	0.1905	0.0154*	0.8404	0.0001*
GD2	29.5 psu	S x T	0.0001*	0.0003*	0.0266*	0.7216	0.1859	0.0007*
GD2	34 psu	S x T	0.0001*	0.0001*	0.0005*	0.0005*	0.0004*	0.0150*
GD1		S x T x Sal	0.2698	0.0017*	0.2798	0.8041	0.1377	0.8175
GD2		S x T x Sal	0.0001*	0.0184*	0.0004*	0.0001*	0.0628	0.0001*
25 psu		S x Sp x T	0.4376	0.0002*	0.0732	0.1945	0.8810	0.0001*
29.5 psu		S x Sp x T	0.6984	0.4486	0.1112	0.1344	0.0276*	0.0002*
34 psu		S x Sp x T	0.0001*	0.0196*	0.0001*	0.2219	0.0012*	0.0001*
No S		Sp x T x Sal	0.0001*	0.0008*	0.0002*	0.0823	0.1520	0.0001*
S		Sp x T x Sal	0.0001*	0.1830	0.1730	0.0029*	0.4053	0.2825
16°C		S x Sp x Sal	0.3157	0.6495	0.4232	0.1867	0.0037*	0.0151*
10°C		S x Sp x Sal	0.4492	0.1358	0.2546	0.2737	0.6765	0.0001*
4°C		S x Sp x Sal	0.0001*	0.0326*	0.001*	0.0133*	0.0795	0.0528
		S x Sp x T x Sal	0.0001*	0.0065*	0.0002*	0.0433*	0.3177	0.0001*

A Permutational Based Multivariate Analysis of Variance (Euclidean distances, 9999 permutations), was used to statistically test two-way, three-way and four-way interaction terms (column 3). Each interaction component (two-way and three-way) was tested for all possible combinations of the factor(s) that were kept constant (column 1–2). Significant p-values are indicated with an asterisk.

Abbreviations: No S, absence of sulphide; S, presence sulphide; T, temperature (16, 10 and 4°C); Sal, salinity (25, 29.5 and 34 psu); Sp, cryptic *Halomonhystera disjuncta* species GD1–3 (previously named *Geomonhystera disjuncta*); MGT, minimum generation time; MEGD, minimum time until first egg(s) deposition; MEMD, minimum time for embryonic development; MJD, minimum time for the development of juveniles into adults; MALS, minimum adult life span; OS, offspring per female at the end of the experiment;/, no test available.

**Table 2:** Estimates of components of variation results of the four-way PERMANOVA on single factors and interaction terms for all analyzed variables.

Single factor and/or interaction terms	MGT	MEGD	MEMD	MJD	MALS	OS
S	2.893	0.526	−0.004	0.870	0.637	183.480
Sp	7.217	0.002	0.311	0.748	3.776	452.880
T	169.230	25.561	10.500	14.894	124.740	93.748
Sal	9.277	1.634	0.106	0.455	0.230	71.800
T x Sal	0.223	0.005	0.046	0.371	0.080	30.517
Sp x Sal	3.163	1.484	0.039	0.197	0.201	25.923
Sp x T	0.384	0.010	0.050	0.101	0.144	3.972
S x Sp	5.498	0.039	2.793	1.826	12.950	160.550
S x Sal	1.744	0.020	0.016	0.214	0.090	13.748
S x T	1.931	0.768	0.005	0.061	0.482	12.609
S x T x Sal	0.139	0.126	0.213	−0.031	0.474	142.200
S x Sp x T	0.210	−0.007	0.026	0.072	0.176	7.579
Sp x T x Sal	0.790	0.090	0.021	0.189	0.082	13.487
S x Sp x Sal	0.232	0.070	0.073	0.151	0.077	20.431
S x Sp x T x Sal	0.696	0.130	0.550	0.197	0.024	44.797
V(Res)	0.269	0.157	0.356	0.432	0.451	20.371

The components of variation was estimated by **PERMANOVA** (Euclidean distances, 9999 permutations) for MGT, minimum generation time; MEGD, minimum time until first egg(s) deposition; MEMD, minimum time for embryonic development; MJD, minimum time for the development of juveniles into adults; MALS, minimum adult life span; OS, offspring per female at the end of the experiment. High values correspond to a high relative importance of the respective factor.

Abbreviations: S, absence/presence of sulphide; T, temperature (16, 10 and 4°C); Sp, cryptic *Halomonhystera disjuncta* species GD1–3 (previously named *Geomonhystera disjuncta*); Sal, salinity (25, 29.5 and 34 psu).

MGT increased with decreasing temperature (T) for all species (Sp; Table 3). When species are compared in presence and absence of sulphide (S), GD2 generally had the shortest MGT at 16°C and at 4°C. Except at cold seep conditions (at a temperature of 4°C, a salinity of 34 psu and in the presence of sulphide), GD1 had a significantly shorter MGT than GD2 (p = 0.0156, Table S1). A significant shorter MGT for GD1, in comparison to GD2-3, was also observed at 10°C (all p-values <0.0018, Table S1). The difference in MGT between species in relation to temperature was underlined by a significant Sp x T interaction (all p-values = 0.0001, Table 1) independent of salinity and sulphide addition.

**Table 3:** Minimum generation time of GD1-3 under changing environmental conditions.

Temperature	Salinity	GD1– No S	GD2 - No S	GD3 - No S	GD1 - S	GD2 - S
16°C	25 psu	9.3±0.6	7.3±0.6	10.3±0.6	9.0±0.0	7.0±0.0
	29.5 psu	9.3±0.6	8.0±0.0	12.0±0.0	9.5±0.6	9.0±0.0
	34 psu	11.25±0.5	9.3±0.5	/	11.3±0.5	10.3±0.4
10°C	25 psu	10.3±0.6	13.7±0.6	13.5±0.6	11.7±0.6	16.0±0.0
	29.5 psu	10.5±0.6	15.7±0.6	19.3±0.6	12.3±0.6	17.7±0.5
	34 psu	12.8±0.5	17.3±0.5	24.3±0.5	14.5±0.6	19.0±0.0
4°C	25 psu	28.3±0.6	25.5±0.6	33.0±0.8	31.3±0.6	28.5±0.5
	29.5 psu	29.7±0.6	28.3±0.5	38.3±0.5	34.3±0.5	33.3±0.5
	34 psu	34.3±0.5	30.3±0.6	43.3±0.6	39.3±0.5	40.5±0.5

Minimum generation time (mean ± standard deviation) of *H. disjuncta* cryptic species (GD1-3) in the absence (No S) and presence of sulphide (S) at different temperatures (16, 10 and 4°C) and salinity (25, 29.5 and 34 psu). No results were obtained for GD3 in the presence of sulphide because these nematodes died within the two first minutes after adding Su. The minimum generation time was depicted from the day we observed the first adult.

Abbreviations: No S, absence of sulphide; S, presence sulphide; GD1-3, cryptic *Halomonhystera disjuncta* species 1–3 (previously named *Geomonhystera disjuncta*).

Increasing the salinity (Sal) resulted in an increase of MGT. The response to higher salinities is more pronounced for GD3 (2–10 days) than for GD1-2 (0–6 days; Table 3), in the absence of sulphide. This observation was supported by a significant Sp x Sal interaction at all temperatures (all p-values <0.0468, Table 1).

The effect of adding sulphide to the cultures resulted in the death of all inoculated GD3 nematodes during the first two minutes and no life-history traits could, therefore, be determined. The addition of sulphide resulted in a significant increase in MGT of 1–10 days for GD1 and GD2 at 10°C and 4°C (all p-values <0.0138, Table S2) at all salinity levels (Table 3).

### Minimum time until first egg(s) deposition (MEGD)

GD3 was the only species showing an ovoviviparous reproduction strategy throughout the whole experiment. Therefore, we did not include GD3 in the MEGD dataset. For the two other species, abiotic factors significantly affected MEGD (four-way PERMANOVA; p = 0.0065, Table 1). Homogeneity of multivariate dispersions was observed (F_35, 89_ = 0.736, p = 0.8453). The estimation of the components of variation revealed that temperature is the most import factor influencing MEGD (Table 2).

**Figure 1:**
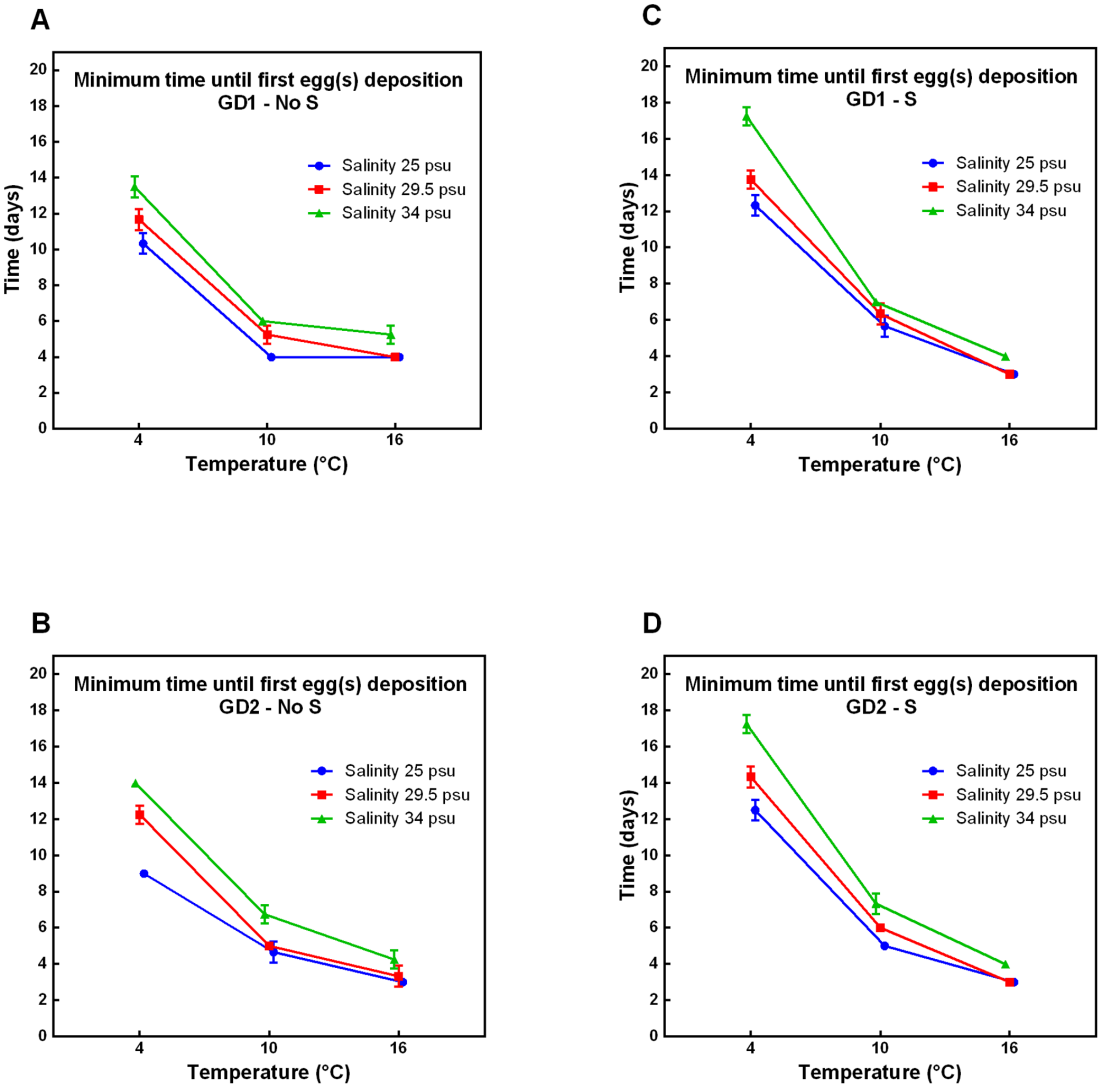
Minimum time until first egg(s) deposition of GD1 and GD2 under changing environmental conditions. Minimum time until first egg(s) deposition in relation to temperature and salinity of cryptic *Halomonhystera disjuncta* species: GD1 (A, C) and GD2 (B, D), in the absence (No S; A, B) and presence (S; C, D) of sulphide. The minimum egg deposition time corresponds to the day after the start of the experiment on which the first egg was observed within the experimental microcosms. Data shown are mean values ±1stdev of 3 or 4 replicates per treatment.

A strong significant increase in MEGD from 10°C to 4°C (all p-values <0.0003, Table S3) was observed for both species (Figure 1). This increase was observed at all salinity levels and in presence/absence of sulphide. Increasing salinity usually resulted in a significant increase in MEGD for both species (p-values in Table S4) and was most pronounced at 4°C for both species, supported by a significant T x Sal interaction term (all p-values <0.0026, Table 1).

The effects of sulphide were heavily dependent on temperature for both species (significant S x T interaction term, all p-values <0.0003 at all salinity levels, Table 1). Addition of sulphide resulted in a significant decrease (all p-values <0.0031, Table S2) in MEGD of GD1 at 16°C, (Figure 1A, 1C) but not of GD2 (Figure 1B, 1C). The observed difference between species was corroborated by significant S x Sp interaction term 16°C at all salinity levels (p-values <0.0383, Table 1). At 10°C, adding sulphide resulted in a significant increase in MEGD of GD1 (all p-values <0.0429, table S2) but not for GD2. At the lowest temperature (4°C), adding sulphide resulted in an increase in MEGD for both species (all p-values <0.0134, Table S2).

### Minimum time for embryonic development (MEMD)

Due to GD3’s ovoviviparous reproduction strategy we were unable to estimate MEMD for this species. Similar to the MEGD, a significant four-way interaction (p = 0.0002, Table 1) and homogeneity of multivariate dispersions were found (F_1,123_ = 0.042, p = 0.8381). The estimates of components of variation revealed that temperature and the interaction term Sp x T were the most important effects (Table 2).

**Figure 2:**
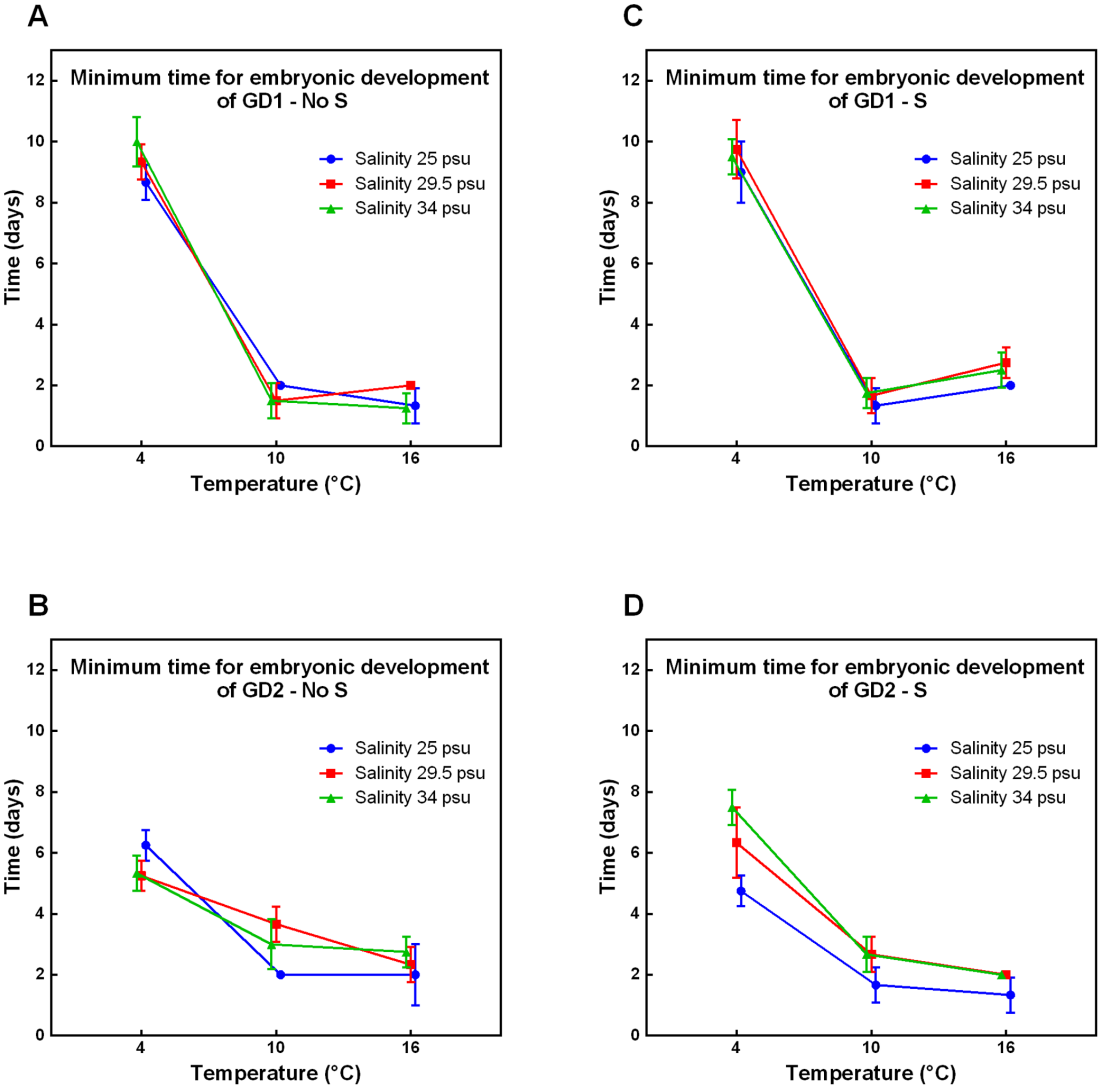
Minimum time for embryonic development of GD1 and GD2 under changing environmental conditions. Minimum time for embryonic development in relation to temperature and salinity of cryptic *Halomonhystera disjuncta* species: GD1 (A, C) and GD2 (B, D), in the absence (No S; A, B) and presence (S; C, D) of sulphide. The minimum time for embryonic development was calculated by subtracting the day we observed the first egg from the day we observed the first juvenile within the experimental microcosms. Data shown are mean values ±1stdev of 3 or 4 replicates per treatment.

Comparable with MEGD, lowering the temperature increased MEMD (Figure 2A–D). Both GD1 and GD2 had a stable MEMD between 16°C and 10°C, while a significant increase was observed at 4°C (all p-values <0.0124, Table S3). This increase was much more pronounced for GD1 than for GD2, underlined by a significant Sp x T interaction effect (all p-values <0.001, Table 1) independent of salinity and sulphide.

Increasing the salinity had no significant effect on MEMD for GD1, but had an opposite effect on MEMD for GD2 at 4°C between different sulphide treatments (Figure 2B, 2D). This observation is supported by a significant S x Sp x Sal interaction component at 4°C (p-value = 0.001, Table 1). The addition of sulphide had no further consistent or opposing effects on MEMD. Interestingly, at bathyal cold seep conditions, GD2 had a significantly shorter MEMD than GD1 (p<0.0029, Table S1).

### Minimum time for the development of juveniles into adults (MJD)

MJD showed a significant four-way interaction effect (p = 0.0433, Table 1) and homogeneity of multivariate dispersions was found (F_43,109_ = 0.870, p = 0.6917). According to the estimates of components of variation, temperature affects MJD the most (Table 2).

**Figure 3:**
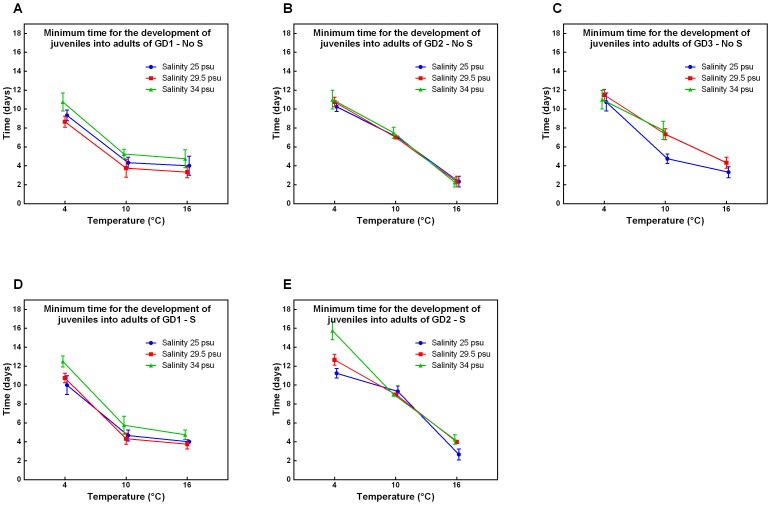
Minimum time for the development of juveniles into adults of GD1-3 under changing environmental conditions. Minimum time for the development of juveniles into adults in relation to temperature and salinity of cryptic *Halomonhystera disjuncta* species: GD1 (A, D), GD2 (B, E) and GD3 (C), in the absence (No S; A–C) and presence (S; D–E) of sulphide. The minimum time for embryonic development was calculated by subtracting the day we observe the first juvenile from the day we observed the first adult within the experimental microcosms. Data shown are mean values ±1stdev of 3 or 4 replicates per treatment. No results were obtained for GD3 in the presence of sulphide because inoculated nematodes died within the first 1–2 minutes after adding Su.

Lowering temperature resulted in a significant increase in MJD (Figure 3A–E) for both GD2 and GD3 independent of other factors (all p-values <0.0189, Table S3). GD1 also showed a significant increase in MJD from 10°C and 16°C to 4°C (all p-values <0.0024, Table S3; Figure 3A, 3B) but not between 16°C to 10°C (all p-values >0.1092, Table S3). This differential effect of temperature changes on species was confirmed by a significant Sp x T interaction effects at all salinity and sulphide levels (all p-values <0.0003, Table 1). Interestingly, GD3 did not produce offspring at 16°C and a salinity of 34 psu. Even though female nematodes had eggs inside the uterus, which developed into juveniles, they were never deposited.

Our data also shows that at 4°C the effect of salinity interacted with the addition of sulphide and was more pronounced for GD2 than for GD1 (significant S x Sp x Sal interaction term, p = 0.0133, Table 1). In addition, at bathyal cold seep conditions GD1 had a significantly shorter MJD compared to GD2 (p = 0.0009, Table S1).

### Minimum adult life span (MALS)

The four-way interaction of MALS was not significant (p = 0.3177, Table 1) and homogeneity of multivariate dispersions (F_41, 99_ = 1.085, p = 0.3638) was observed. Some data points are missing because in some treatments we stopped the experiment before any mortality of inoculated nematodes occurred.

**Figure 4:**
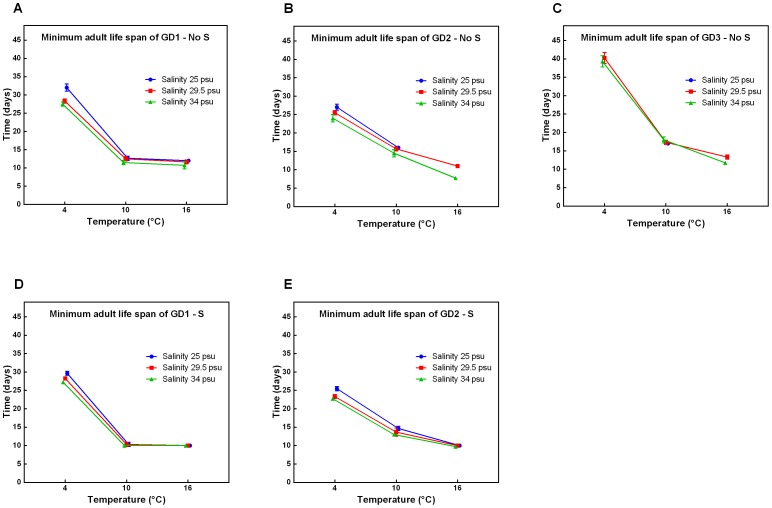
Minimum adult life span under changing environmental conditions. Minimum adult life span in relation to temperature and salinity of cryptic *Halomonhystera disjuncta* species: GD1 (A, D), GD2 (B, E) and GD3 (C), in the absence (No S; A–C) and presence (S; D–E) of sulphide. The minimum adult life span corresponds to the day after the start of the experiment on which the first inoculated nematode died. Data shown are mean values ±1stdev of 3 or 4 replicates per treatment. No results were obtained for GD3 in the presence of sulphide because inoculated nematodes died within the first 1–2 minutes after adding Su. Some data points are missing because the experiment was stopped before the death of any of the inoculated nematodes.

A decrease in temperature generally resulted in a significant increase in MALS for all species (Figure 4). However, no significant increase was observed for GD1 from 16°C to 10°C (all p-values >0.1167, Table S3), in contrast with GD2 and GD3 (all p-values <0.0010, Table S3). At 4°C, in the absence of sulphide, GD2 and GD3 had the shortest and largest MALS, respectively. This difference between species was supported by a significant Sp x T interaction effect at all salinity and sulphide levels (all p-values <0.0002, Table 1).

Addition of sulphide resulted in the death of all inoculated GD3 nematodes within minutes. For both other species the addition of sulphide commonly decreased MALS. At 4°C and at bathyal cold seep conditions, GD1 had a higher MALS than GD2 (all p-values <0.0023, Table S1, Figure 4D–E). Sulphide-induced mortality decreased with decreasing temperature: in the presence of sulphide and at a temperature of 16°C, ca. 99% of both inoculated and new adults of GD1 and GD2 died after one generation. However, this high mortality rate was decreased to ca. 50% at 10°C and was negligible at 4°C.

### Offspring per female (OS)

Variances were homogeneous (F_44, 112_ = 0.526, p = 0.9931) and a significant four-way interaction effect was found (p = 0.0001, Table 1). Estimates of components of variation revealed that most factors and interactions had an effect on the OS (Table 2) with temperature being the most important one. Consequently, sub-datasets were analyzed to better understand effects of single factors and their two- and three-way interactions.

**Figure 5:**
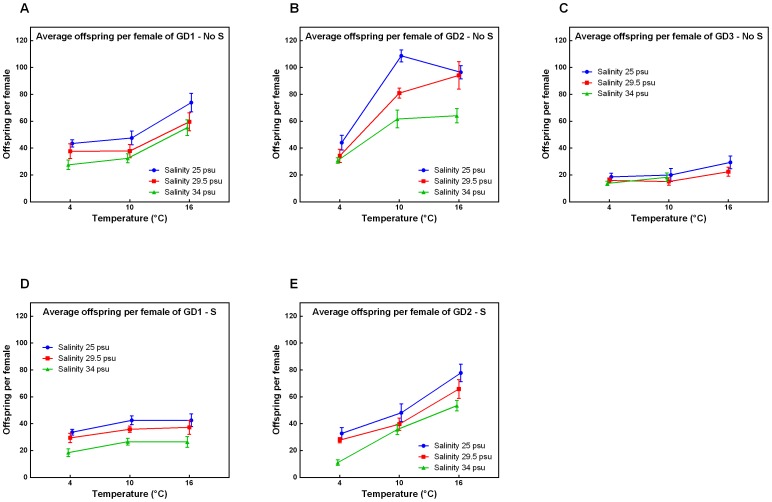
Offspring per female of GD1-3 under changing environmental conditions. Offspring per female in relation to temperature and salinity of cryptic *Halomonhystera disjuncta* species: GD1 (A, D), GD2 (B, E) and GD3 (C), in the absence (No S; A–C) and presence (S; D–E) of sulphide. The offspring corresponds to the amount of eggs, juveniles and new adults at the end of the experiment, averaged across six females. Data shown are mean values ±1stdev of 3 or 4 replicates per treatment. No results were obtained for GD3 in the presence of sulphide because inoculated nematodes died within the first 1–2 minutes after adding sulphide.

All cryptic species had the highest OS at 16°C in the absence of sulphide and at a salinity of 25 psu (except for GD2, Figure 5), and the lowest OS at 4°C in the presence of sulphide and at a salinity of 34 psu. A reduction in OS was observed for all species with decreasing temperature, increasing salinity and addition of sulphide. However, the declination in OS for GD2 was clearly more influenced by temperature than for GD1 and GD3 as was supported by a significant Sp x T interaction component (all p-values = 0.0001, Table 1). Variation in OS, caused by temperature and salinity, was much lower in GD3 in the absence of sulphide (p-value _Sp x T x Sal_ = 0.0001, Table 1), compared to the two other species. However, no offspring was observed at 16°C and a salinity of 34 psu. Generally, adding sulphide resulted in a reduction in OS for both GD1 and GD2. Furthermore, GD1 produced significantly more OS than GD2 at bathyal cold seep conditions (p = 0.006, Table S1).

## Discussion

The success of nematodes in marine environments relates to their ability to adapt to changing environmental conditions [54], [55]. Because the three cryptic *H. disjuncta* species commonly occur on decaying macroalgae in the high intertidal, they are regularly exposed to short- and long-term fluctuations in abiotic factors such as salinity and temperature. Moreover, they can be exposed to hydrogen sulphide produced by sulphate reducing bacteria thriving on rotting macroalgae [56]. In summary, the three species were expected to show wide tolerances to the abiotic factors tested in this study. In our experiments, temperature had the strongest impact on life-history traits of all cryptic species. The minimum generation time and adult life span increased with decreasing temperature, which is most likely caused by lower metabolic rates at lower temperatures [57]. Faster generation times at higher temperatures shorten the vulnerable period of the embryo, thus preventing embryonic deformities and/or arrest [54], and allow species to rapidly increase in numbers during favorable episodes, a common feature of opportunistic species [58]. *Halomonhystera disjuncta* is indeed an opportunistic species capable of quickly colonizing suitable patches such as new algal deposits [35]. Salinity, on the other hand, had a relatively minor impact on the life-history traits studied here. The salinity and temperature ranges tested in our study reflect fluctuations in their natural habitat and indicate a strong capacity for adaptation to natural fluctuations, which is consistent with previous studies on other marine nematodes from very similar intertidal habitats [59], [60].

### Cryptic *H. disjuncta* species are not ecologically equivalent

Co-occurrence of cryptic species raises questions regarding their ecological similarity and the mechanisms influencing their coexistence [15], [19]. The three cryptic species studied here have a very similar morphology, have sympatric occurrences and all thrive on the same habitat, i.e. decaying macroalgae in the Westerschelde estuary. We may, therefore, expect that they have evolved comparable adaptations to similar environmental conditions (equalizing mechanism). The general response to temperature and salinity, i.e. an increase in time for life-history traits and a decrease in number of offspring with lower temperatures and higher salinities, was indeed similar in all cryptic species and points towards ecological equivalence. However, our results also uncovered specific life-history responses to different abiotic factors. Life-history traits of GD1 were more stable in the temperature interval between 10°C and 16°C than those of GD2. Interestingly, GD3 did not survive in the presence of sulphides, whereas GD1 and GD2 not only survived but also reproduced and developed normal offspring for at least one generation. These results reveal that cryptic species are not completely ecologically equivalent. Our results might be partly biased because a single gravid female was used to generate the monospecific cultures. Nevertheless, the observed differential effects of abiotic factors on life-history traits, may provide important stabilizing mechanisms facilitating the coexistence of cryptic species [9].

In addition to its sensitivity to sulphides, the most obvious difference between GD3 and the other two cryptic species was its ovoviviparous reproduction strategy. Intrauterine egg hatching (ovoviviparity) allows a more secure survival and early development of offspring and can be an adaptation strategy in toxic environmental conditions [61]. The low number of GD3 offspring compared to GD1 and GD2 suggests a trade-off between the reproductive strategy and fecundity. This is further supported by the observation that the fecundity of GD3 was less affected by changes in salinity and temperature than that of GD1 and GD2. In addition, the minimum adult life span was always the highest for GD3 at all temperatures. The comparatively smaller impact of temperature on GD3 could also in part explain its occurrence throughout the year and its high relative abundance in winter in the Westerschelde estuary [23]. In contrast to GD3, GD2 was often absent in winter but had the highest relative abundance in spring [23]. Our experimental data revealed that GD2 had the fastest minimum generation time at 16°C and the highest number of offspring at 10°C and 16°C. In spite of this, its number of offspring was more sensitive to temperature changes. These observations may explain the high relative abundance of GD2 in spring and its disappearance in winter.

Differential responses of nematodes to abiotic factors can also affect interspecific interactions [27], [62] and dispersal capacities [26], which could in turn facilitate coexistence. Recently, significant differences in resource use among sympatric cryptic nematode species have been demonstrated, and other potentially discriminating biotic/abiotic factors, such as differential susceptibility to predation and competitive interaction with other taxa, should be taken into account to fully grasp the coexistence and spatial/temporal distribution patterns of these cryptic species because the small morphological differences between GD1-3 [34] may not be sufficient to predict ecological segregation [63].

### GD1 and the Nordic seep nematode *Halomonhystera hermesi* share a common ancestor

It is often suggested that current deep-sea biodiversity has largely resulted from recurrent invasions from bathyal and abyssal depths followed by speciation [64]. The existence of morphologically and/or genetically close relatives from intertidal and deep-sea environments [65] tends to support this contention. In an evolutionary context, understanding the adaptations which have allowed deep-sea colonization is an important prerequisite. GD1 is phylogenetically most closely related to the Nordic seep nematode *Halomonhystera hermesi* [44] and, therefore, shares a most recent common ancestor with *H. hermesi.*

In the reduced environment of the Håkon Mosby mud volcano (HMMV), organisms encounter stress levels which selectively favor sulphide tolerant species [66]. In view of the 100% mortality of GD3 in the sulphide treatments, it is very unlikely that this species successfully colonized the HMMV. It must, however, be mentioned that GD3 did survive under lower concentrations of sodium thiosulphate and sodium sulphide (up to 0.25 µM) in a preliminary test. Such a mild exposure could increase resistance to subsequent higher doses (hormesis) of the same or different stressors [67]. Nevertheless, GD3 was unable to cope with high concentrations of sulphides. GD1 and GD2, by contrast, survive and produce offspring at the sulphide concentrations tested here. Such a high sulphide tolerance may be an advantage for colonizing reduced environments [66]. Species living in sulphidic environments often have ecto- or endosymbionts which could either serve as a food source and/or play a role in sulphur detoxification [68], [69]. In addition, sulphur inclusions could temporarily reduce the toxic effect of H_2_S [70]. However, no signs of bacterial symbionts in *H. hermesi* were detected [45], and we did not observe sulphur inclusions in GD1-2. Interestingly, however, low temperature had a positive effect on the survival of GD1 and GD2 (but not GD3) under sulphide exposure. Similarly, low temperatures reduced the mortality rate of *H. disjuncta* subjected to toxic chromium concentrations [40]. Hence, the low temperatures in the deep sea may have been an important factor enabling *Halomonhystera* to cope with otherwise toxic sulphide concentrations.

Our results further reveal that GD1 had a higher number of offspring at bathyal cold seep conditions (salinity of 34 psu – temperature of 4°C – presence of sulphides) than GD2. In addition, GD1 had the fastest minimum generation time and a longer minimum life span than GD2 at these conditions. This implies that GD1 is more resistant to a combination of sulphides, low temperature and higher salinities, and is in agreement with the close phylogenetic relationship between GD1 and *H. hermesi*, supporting the idea that it could have successfully colonized Nordic seeps.

## Conclusion

We have shown that life-histories of cryptic were differentially affected by changes in temperature, salinity and presence of sulphides. These observed differences imply that closely related cryptic *H. disjuncta* species are not necessarily ecologically equivalent and that abiotic factors can strongly impact their life-history traits, which in turn can affect their coexistence. Our results further indicate that *Halomonhystera hermesi* and GD1 share a common ancestor as the latter appears to be more resistant to bathyal seep conditions compared to other cryptic species. The observed limited ecological equivalence among cryptic species may have important repercussions for our understanding of the link between biodiversity and ecosystem functioning [71].

## Supporting Information

Table S1 **P-values of pairwise comparisons between species with the same treatment.** PERMANOVA (Euclidean distances, 9999 permutations) was used for the pairwise comparisons. The table part between double lines contains the variable factor species (GD1-3) on which we performed pairwise comparisons for the variables in column 2. Row 1–2 and column 1 contain fixed factor levels for the respective column and row. Significant p-values are indicated with an asterisk.
(DOCX)

Table S2 **P-values of pairwise comparisons between treatments which differ in presence or absence of sulphide.** PERMANOVA (Euclidean distances, 9999 permutations) was used for the pairwise comparisons. The table part between double lines contains the variable factor sulphide (sulphide present = S, sulphide absent = No S) on which we performed pairwise comparisons for the variables in column 2. Significant p-values are indicated with an asterisk.
(DOCX)

Table S3 **P-values of pairwise comparisons between treatments which differ in one level of the factor temperature.** PERMANOVA (Euclidean distances, 9999 permutations) was used for the pairwise comparisons. The table part between double lines contains the variable factor temperature (16, 10 and 4°C) on which we performed pairwise comparisons for the variables in column 2. Significant p-values are indicated with an asterisk.
(DOCX)

Table S4 **P-values of pairwise comparisons between treatments which differ in one level of the factor salinity.** PERMANOVA (Euclidean distances, 9999 permutations) was used for the pairwise comparisons. The table part between double lines contains the variable factor salinity (25, 29.5 and 34 psu) on which we performed pairwise comparisons for the variables in column 2. Significant p-values are indicated with an asterisk.
(DOCX)

Data S1 **Complete overview of the raw data as was collected during the experiment.**
(XLSX)
