# Supplementary material for: Differences in Life-Histories Refute Ecological Equivalence of Cryptic Species and Provide Clues to the Origin of Bathyal Halomonhystera (Nematoda)
Source: PLoS One. 2014 Nov 10;9(11):e111889. doi: 10.1371/journal.pone.0111889 (PMC4226489; doi:10.1371/journal.pone.0111889)
Supplement: Table S1 — P-values of pairwise comparisons between species with the same treatment. PERMANOVA (Euclidean distances, 9999 permutations) was used for the pairwise comparisons. The table part between double lines contains the variable factor species (GD1-3) on which we performed pairwise comparisons for the variables in column 2. Row 1–2 and column 1 contain fixed factor levels for the respective column and row. Significant p-values are indicated with an asterisk. (DOCX) [file pone.0111889.s001.docx]

**Table S1: P-values of pairwise comparisons between species with the same treatment.** PERMANOVA (Euclidean distances, 9999 permutations) was used for the pairwise comparisons. The table part between double lines contains the variable factor species (GD1-3) on which we performed pairwise comparisons for the variables in column 2. Row 1-2 and column 1 contain fixed factor levels for the respective column and row. Significant p-values are indicated with an asterisk.

|  |  | **Temperature** | 16°C | | | | | |  | 10°C | | | | | |  | 4°C | | | | | |
| --- | --- | --- | --- | --- | --- | --- | --- | --- | --- | --- | --- | --- | --- | --- | --- | --- | --- | --- | --- | --- | --- | --- |
|  |  | **Salinity** | 25 psu | | 29.5 psu | | 34 psu | |  | 25 psu | | 29.5 psu | | 34 psu | |  | 25 psu | | 29.5 psu | | 34 psu | |
| **Presence of sulphide** | **Variable** | **Species** | GD1 | GD2 | GD1 | GD2 | GD1 | GD2 |  | GD1 | GD2 | GD1 | GD2 | GD1 | GD2 |  | GD1 | GD2 | GD1 | GD2 | GD1 | GD2 |
| No S | MGT | GD2 | 0.0112* |  | 0.0193* |  | 0.0011* |  |  | 0.0018* |  | 0.0004* |  | 0.0002* |  |  | 0.0014* |  | 0.0186* |  | 0.0005* |  |
|  |  | GD3 | 0.1031 | 0.0024* | 0.0012* | / |  |  |  | 0.0012* | 0.7208 | 0.0001* | 0.0017* | 0.0001* | 0.0001* |  | 0.0005* | 0.0001* | 0.0001* | 0.0001* | 0.0001* | 0.0002* |
|  | MEGD | GD2 | / |  | 0.1111 |  | 0.0292* |  |  | 0.1171 |  | 0.4396 |  | 0.0257* |  |  | 0.0072* |  | 0.2036 |  | 0.2081 |  |
|  | MEMD | GD2 | 0.3691 |  | 0.3738 |  | 0.0042* |  |  | / |  | 0.0055* |  | 0.0251* |  |  | 0.0019* |  | 0.0005* |  | 0.0008* |  |
|  | MJD | GD2 | 0.0688 |  | 0.0994 |  | 0.0045* |  |  | 0.0018* | | 0.0022* |  | 0.0017* |  |  | 0.0789 |  | 0.0040* |  | 0.7457 |  |
|  |  | GD3 | 0.3688 | 0.1045 | 0.0983 | 0.0140* |  |  |  | 0.3484 | 0.0004* | 0.0031* | 0.3823 | 0.0051* | 0.6699 |  | 0.0777 | 0.3834 | 0.0017* | 0.1019 | 0.7525 | / |
|  | MALS | GD2 | / |  | 0.1171 |  | 0.0013* |  |  | 0.0379* |  | 0.0004* |  | 0.0017* |  |  | 0.0009* |  | 0.0017* |  | 0.0023* |  |
|  |  | GD3 | / | / | 0.0258* | 0.0020* | 0.1094 | 0.0010* |  | 0.0249* | / | 0.0001* | 0.0225* | 0.0001* | 0.0019* |  | / | / | 0.0001* | 0.0001* | 0.0001* | 0.0002* |
|  | OS | GD2 | 0.0087* |  | 0.0086* |  | 0.0664 |  |  | 0.0002* |  | 0.0001* |  | 0.0004* |  |  | 0.8821 |  | 0.4071 |  | 0.2220 |  |
|  |  | GD3 | 0.0005* | 0.0001* | 0.0010* | 0.0004* | 0.0002* | 0.0001* |  | 0.0006* | 0.0001* | 0.0011* | 0.0001* | 0.0010* | 0.0002* |  | 0.0003* | 0.0003* | 0.0004* | 0.0006* | 0.0015* | 0.0005* |
| S | MGT | GD2 | / |  | 0.1308 |  | 0.0327* |  |  | 0.0002* |  | 0.0003* |  | 0.0001* |  |  | 0.0016* |  | 0.0724 |  | 0.0156* |  |
|  | MEGD | GD2 | / |  | / |  | / |  |  | 0.1117 |  | 0.3671 |  | 0.2874 |  |  | 0.7223 |  | 0.2156 |  | 1 |  |
|  | MEMD | GD2 | 0.1177 |  | 0.0256* |  | 0.1345 |  |  | 0.5149 |  | 0.1041 |  | 0.0752 |  |  | 0.0001* |  | 0.0086* |  | 0.0029* |  |
|  | MJD | GD2 | 0.0155* |  | 0.3565 |  | 0.2064 |  |  | 0.0007* |  | 0.0003* |  | 0.0018* |  |  | 0.0781 |  | 0.0043* |  | 0.0009* |  |
|  | MALS | GD2 | / |  | / |  | 0.3603 |  |  | 0.0008* |  | 0.0115* |  | / |  |  | 0.0002* |  | 0.0002* |  | 0.0001* |  |
|  | OS | GD2 | 0.0017* |  | 0.0005* |  | 0.0003* |  |  | 0.2524 |  | 0.2691 |  | 0.0109* |  |  | 0.7759 |  | 0.4945 |  | 0.006* |  |

Abbreviations: No S, absence of sulphide; S, presence sulphide; GD1-3, cryptic *Halomonhystera disjuncta* species 1 – 3 (previously named *Geomonhystera disjuncta*); MGT, minimum generation time; MEGD, minimum time until first egg(s) deposition; MEMD, minimum time for embryonic development; MJD, minimum time for the development of juveniles into adults; MALS, minimum adult life span; OS, offspring per female at the end of the experiment; /, no test available.
